# Systemic inflammation in 222.841 healthy employed smokers and nonsmokers: white blood cell count and relationship to spirometry

**DOI:** 10.1186/1617-9625-10-7

**Published:** 2012-05-21

**Authors:** José Antonio Fiz Fernández, Josép Morera Prats, José Vicente Monsonis Artero, Alberto Calvo Mora, Anna Vazquez Fariñas, Anna Espinal, José Antonio Gelpi Méndez

**Affiliations:** 1 Hospital Universitari Germans Trias i Pujol, Servicio de Neumología, Planta 8. Carretera del Canyet s/n, Badalona, Barcelona, 08916, Spain; 2 Sociedad de Prevención de Ibermutuamur, Argos 4-6, Madrid, 28037, Spain; 3 Servei d’statística Aplicada, Universitat Autónoma de Barcelona, Bellaterra (Cerdanyola del valles), 08193, Spain

## Abstract

**Background:**

Smoking has been linked to low-grade systemic inflammation, a known risk factor for disease. This state is reflected in elevated white blood cell (WBC) count.

**Objective:**

We analyzed the relationship between WBC count and smoking in healthy men and women across several age ranges who underwent preventive medical check-ups in the workplace. We also analysed the relationship between smoking and lung function.

**Methods:**

Cross-sectional descriptive study in 163 459 men and 59 382 women aged between 16 and 70 years. Data analysed were smoking status, WBC count, and spirometry readings.

**Results:**

Total WBC showed higher counts in both male and female smokers, around 1000 to 1300 cell/ml (*t* test, *P* < 0.001). Forced expiratory volume in 1 second (FEV_1_%) was higher in nonsmokers for both sexes between 25 to 54 years (*t* test, *P* < 0.001). Analysis of covariance showed a multiple variable effect of age, sex, smoking status, body mass index on WBC count. The relationship between WBC blood count and smoking status was confirmed after the sample was stratified for these variables. Smokers with airway obstruction measured by FEV_1_% were found to have higher WBC counts, in comparison to smokers with a normal FEV_1_% among similar age and BMI groups.

**Conclusions:**

Smoking increases WBC count and affects lung function. The effects are evident across a wide age range, underlining the importance of initiating preventive measures as soon as an individual begins to smoke.

## Background

Since the 1970s smoking has been linked to low-grade systemic inflammation as reflected in elevated white blood cell (WBC) count [1-3], a well-established predictor of such serious health events as myocardial infarction [4], cancer [5], and chronic obstructive pulmonary disease [6]. Experiments in animals have demonstrated that tobacco smoke promotes leukocyte transit from bone marrow to small pulmonary vessels and that the effect on alveolar walls favours the development of pulmonary emphysema [7]. However, WBC count is also influenced by a range of acute and chronic infectious and inflammatory processes that are difficult to screen for in population studies [8,9]. We therefore sought to describe the behaviour of WBC count in subjects known to be free of health conditions that could affect the results of laboratory tests, in the interest of providing data that would be useful for comparison with studies of the effect of tobacco smoking in either healthy or diseased populations.

To that end we analysed data available for a healthy employed population of men and women in a wide age range. Health vigilance programmes in Spain require that individuals undergoing preventive medical check-ups in the workplace be free of any acute condition. When a worker is found to have an acute illness or an exacerbation of a chronic condition, the examination is postponed until the person has recovered. Therefore, the worker is known to be healthy at the time blood samples are taken. Our hypothesis was that laboratory results for this healthy working population would be less influenced by nonsmoking-related factors that might affect WBC count in a random population-based sample.

To confirm that smoking by itself leads to a state of sustained systemic inflammation, we conducted a cross-sectional study of WBC count in healthy male and female smokers and nonsmokers across several age ranges who underwent preventive medical check-ups in the workplace, analysing the relationship between smoking and WBC count. Additionally, we sought to confirm a relationship between WBC count and declining lung function.

## Material and methods

### Design and setting

For this cross-sectional descriptive study we gathered clinical data from the 2009 records of Ibermutuamur Society for Preventive Medicine (Sociedad de Prevención de Ibermutuamur, Madrid). In 2009, this occupational health insurer covered 518 587 workers in 29 845 companies located throughout Spain. A total of 380 353 individuals underwent a regularly scheduled health check-up as part of the insurer’s vigilance programme.

### Study population

Data for 163 459 men and 59 382 women, all white, between the ages of 16 and 70 years were examined between 1 January and 31 December 2009. This population sample, accounting for 58.6% of the insured workers attending the preventive medical check-up that year, met all the requirements for inclusion. Thus, morning blood samples were taken from workers who had fasted for at least 12 hours and who had been told not to smoke before the check-up. Subjects were allowed to have taken any prescribed medications with water.

### Data collection and variables

An exhaustive medical history included the variables of interest for this study: smoking status (current smoker or nonsmoker), WBC count in peripheral blood (cells/ml), and spirometry readings (forced vital capacity [FVC], forced expiratory volume in 1 second [FEV_1_], and the ratio of FEV_1_ to FVC). The data belong to the health vigilance programme of the insurer, which is responsible for safe storage.

Other variables on record were anthropometric data (weight, height, body mass index [BMI] calculated as kg/m^2^). To calculate BMI weight and height were measured (SECA-711 220, Class III scale; Medizinische Waagen und Messsysteme, Hamburg, Germany) with the worker wearing light clothing and no shoes. To reduce clothing weight further, objects were removed from pockets and belts and other accessories were taken off. These measurements were taken in the morning after urination and fasting. The scale was checked and calibrated periodically by an independent company (Reimedical, SL; Madrid, Spain).

For spirometry, examiners used a Spirolab II RS232 device (Medical International Research, Rome, Italy) as part of the routine check-up. To ensure quality of measurements, the equipment was inspected by the health insurer’s staff and calibrated by an independent company, following the guidelines of the Spanish Society of Pulmonology and Thoracic Surgery (SEPAR) [10]. The worker was encouraged by the examiner to take a deep breath and then exhale as forcefully as possible. The highest values for FVC and FEV_1_ were recorded. The results were compared with reference values for a Spanish population, using the most recently updated tables [11].

All blood samples were analysed by the same laboratory (Megalab, SA, Madrid) using the same automated cell counter (Cell-Dyn Sapphire, Abbott Laboratories, Abbott Park, IL, USA).

### Statistical analysis

After verifying normality of distribution, we subjected the data set to *t* test analysis or variance analysis (ANOVA) to compare differences between groups, followed by analysis of covariance (ANCOVA) to find confounding variables. Medians were compared using nonparametric tests as appropriate for the variable. Smokers and nonsmokers were compared by sex and by age and BMI strata. Results of ANOVA were expressed as mean (SD) and 95% confidence interval (CI); results of ANCOVA were expressed as mean (SEM). *P* values less than 0.05 were considered statistically significant. Statistics were compiled and analysed with GraphPad Quick Calcs software (San Diego, CA, USA).

## Results

The characteristics of the population of 163 459 men and 59 382 women are shown in Table 1, by smoking habit. The mean (SD) age was 39.6 (10.6) years, with significant differences between smokers and nosmokers (*P < 0.05*). Among males the BMI of non smokers was statistically higher than that of smokers (by less than 1 kg/m3) among all age groups, while among females, BMI was statistically higher among non smokers (by less than 1 kg/m3) (Table 2).

**Table 1:** Anthropometric values of the population of healthy workers

	**Male**			**Female**		
	**Nonsmokers**	**Smokers**	**All**	**Nonsmokers**	**Smokers**	**All**
	**(n = 78 306)**	**(n = 85 153)**	**(n = 163 459**	**(n = 27 883)**	**(n = 31 499)**	**(n = 59 382)**
**Age (years)**	41.7 (41.59–41.74)	38.29 (38.22–38.36)*	39.91 (39.85–39.96)	40.84 (40.71–40.96)	36.70 (36.60–36.80)*	38.64 (38.56–38.73)
**Weight (kg)**	82.6 (82.48–82.67)	80.94 (80.95–81.04)*	81.78 (81.66–81.79)	64.71 (64.57–64.85)	63.41 (63.28–63.54)*	64.02 (63.92–64.12)
**Height (m)**	1.735 (1.735–1.736)	1.742 (1.741–1.742)	1.739 (1.738–1.739)	1.613 (1.612–1.614)	1.625 (1.624–1.625)*	1.619 (1.618–1.620)
**BMI (kg/m**^**2**^**)**	27.4 (27.36–27.41)	26.65 (26.62–26.68)*	27.00 (26.98–27.02)	24.89 (24.84–24.95)	24.02 (23.97–24.07)	24.43 (24.39–24.47)

Values are means and 95% confidence intervals.

* Statistically significant differences between smokers and nonsmokers (*t* test for independent groups, *P* < 0.05).

*BMI* Body Mass Index.

**Table 2:** BMI for healthy workers

	**Male**		**Female**	
**Age (years)**	**Nonsmokers**	**Smokers**	**Nonsmokers**	**Smokers**
	**(n = 78 306)**	**(n = 85 153)**	**(n = 27 883)**	**(n = 31 499)**
**16–24**	24.85 (24.73–24.97)*	24.23 (24.14–24.33)	22.99 (22.79–23.20)	22.86 (22.70–23.02)
**25–34**	26.30 (26.24–26.35)*	26.02 (25.97–26.07)	23.54 (23.45–23.64)	23.34 (23.26–23.41)
**35–44**	27.43 (27.38–27.48)*	27.11 (27.06–27.16)	24.71 (24.61–24.81)*	24.21 (24.13–24.30)
**45–54**	28.21 (28.16–28.26)*	27.53 (27.47–27.60)	25.89(25.79–25.99)*	25.23 (25.12–25.34)
**55–64**	28.55 (28.48–28.62)*	27.68 (27.58–27.78)	26.90 (26.74–27.05)*	25.83 (25.59–26.03)
**>64**	28.23 (27.83–28.62)	27.72 (27.02–28.42)	26.78 (26.08–27.49)	24.69 (21.44–27.93)

Values are means and 95% confidence intervals.

* Statistically significant differences between smokers and nonsmokers, for both males and females (*t* test for independent groups, *P* < 0.001).

BMI*: Body Mass Index (kg/m*^*2*^*).* n: *Subjects number****.***

**Figure 1:**
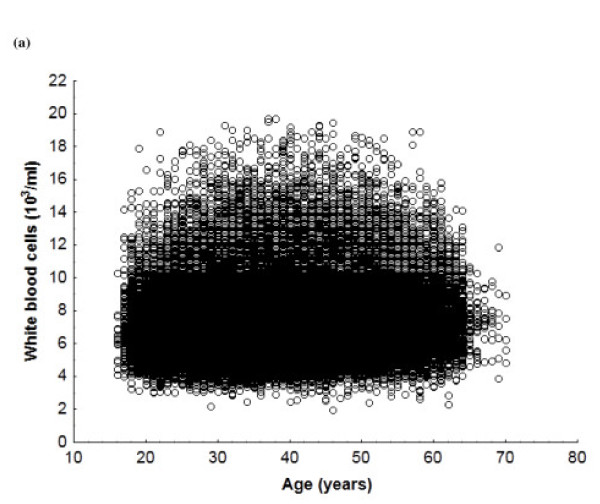
White blood cell count in a) male smokers and b) male nonsmokers.

**Figure 2:**
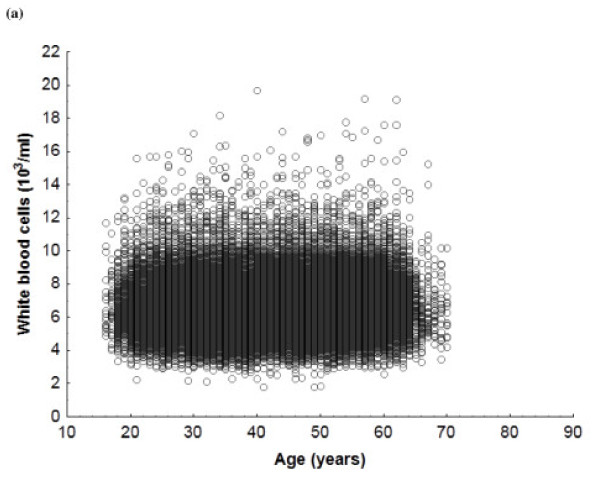
White blood cell count in a) female smokers and b) female nonsmokers.

Total WBC counts were higher in smokers of both sexes by around 1180 cells/ml (ANOVA, *P* < 0.001; *t* test for independent data, *P* < 0.001). ANOVA confirmed age (in six ranges) and smoking (in two categories) as independently associated with WBC count in men and women (Table 3). Men have around 1000 cells/ml more than women. Scatterplots of the point distribution of WBC counts for the population show that higher counts were found in male and female smokers (Figures 1 and 2).

**Table 3:** White blood cell counts (cells/ml) for healthy workers

	**Male**			**Female**		
**Age (years)**	**Nonsmokers**	**Smokers**	**All**	**Nonsmokers**	**Smokers**	**All**
	**(n = 78 306)**	**(n = 85 153)**	**(n = 163 459)**	**(n = 27 883)**	**(n = 31 499)**	**(n = 59 382)**
**16–24**	6555 (6509–6601)*	7296 (7253–7339)	7010 (6978–7042)1	6771 (6681–6861)*	7391 (7317–7466)	7168 (7109–7226)
**25–34**	6456 (6434–6437)*	7569 (7546–7592)	7118 (7100–7135)	6523 (6485–6561)*	7292 (7257–7327)	6995 (6968–7021)
**35–44**	6436 (6416–6455)*	7927 (7902–7953)	7244 (7226–7261)	6368 (6333–6404)*	7441 (7401–7480)	6973 (6944–7001)
**45–54**	6512 (6491–6532)*	8124 (8092–8155)	7245 (7225–7265)	6164 (6132–6196)*	7421 (7372–7471)	6699 (6670–6729)
**55–64**	6600 (6572–6629)*	7914 (7864–7965)	7076 (7048–7103)	5989 (5939–6040)*	7235 (7126–7343)	6313 (6264–6363)
**>64**	6518 (6358–6680)	7410 (7132–7688)	6744 (6600–6888)	5987 (5677–6296)	6821 (5489–8153)	6058 (5758–6359)

Values are means and 95% confidence intervals.

* Statistically significant differences between smokers and nonsmokers, for both males and females (*t* test for independent groups, *P* < 0.001).

n: Subject number.

The analysis of spirometric findings by smoking status, age and sex showed that FEV_1_% (Forced expiratory volume in one second in percentage respect to normal values) was higher in male nonsmokers from the age of 25 years onward and in female nonsmokers in the range of ages from 25 years to 54 years (Table 4). The findings were similar for the FEV_1_/FVC ratio and FVC% (Forced vital capacity expressed as a percentage respect to normal values) (Table 5).

**Table 4:** **Forced expiratory volume in 1 second (FEV**_**1**_**and FEV**_**1**_**%) in active workers**

	**Male**		**Female**	
**Age (years)**	**Nonsmokers**	**Smokers**	**Nonsmokers**	**Smokers**
	**(n=78 306)**	**(n=85 153)**	**(n=27 883)**	**(n=31 499)**
**16–24**				
**FEV**_**1**_**(l)**	4.245 (4.225–4.265)	4.219 (4.203–4.234)	3.075 (3.050–3.100)	3.083 (3.065–3.101)
**FEV**_**1**_**%**	98.5 (98.1–98.9)	98.3 (98.0–98.6)	94.3 (93.6–94.9)	94.6 (94.1–95.1)
**25–34**				
**FEV**_**1**_**(l)**	4.170 (4.160–4.1807)	4.140 (4.132–4.148)	3.073 (3.063–3.084)	3.067 (3.060–3.075)
**FEV**_**1**_**%**	100.4 (100.2–100.6)*	99.3 (99.1–99.5)	98.6 (98.3–98.9)*	97.8 (97.5–97.9)
**35–44**				
**FEV**_**1**_**(l)**	3.936 (3.928–3.945)	3.832 (3.824–3.839)	2.918 (2.908–2.929)	2.861 (2.851–2.870)
**FEV**_**1**_**%**	102.3 (102.1–102.5)*	99.4 (99.2–99.6)	102.6 (102.2–102.9)*	100.1 (99.8–100.4)
**45–54**				
**FEV**_**1**_**(l)**	3.588 (3.580–3.596)	3.388 (3.379–3.397)	2.630 (2.620–2.639)	2.536 ( 2.525–2.548)
**FEV**_**1**_**%**	102.6 (102.3–102.8)*	97.0 (96.7–97.2)	103.9 (103.5–104.2)*	98.7 (98.3–99.1)
**55–64**				
**FEV**_**1**_**(l)**	3.142 (3.131–3.153)*	2.917 (2.901–2.932)	2.305 (2.290–2.320)	2.219 (2.186–2.239)
**FEV**_**1**_**%**	100.8 (100.4–101.1)	93.5 (93.0–93.9)	103.6 (103.0–104.2)	98.6 (97.5–99.8)
**>64**				
**FEV**_**1**_**(l)**	2.887 (2.825–2.949)	2.576 (2.475–2.677)	2.051 (1.955–2.14)	1.851 (1.538–2.163)
**FEV**_**1**_**%**	98.9 (97.7–100.8)*	90.3 (86.8–93.7)	104.5 (99.9–109)	86.5 (79.1–93.9)

Values are means and 95% confidence intervals.

* Statistically significant differences in FEV_1_% between smokers and nonsmokers for males and females (*t* test for independent groups, *P* < 0.001).

FEV_1_(l): *Forced expiratory volume in 1 second in liters*.

FEV_1_%: *Forced expiratory volume in one second in percentage respect to normal values.*

**Table 5:** **Forced vital capacity (FVC) and FVC% and the ratio FEV**_**1**_**to FVC (FEV**_**1**_**/FVC%) in healthy active workers**

	**Males**		**Females**	
**Age (years)**	**Nonsmokers(n=78 306)**	**Smokers(n=85 153)**	**Nonsmokers(n=27 883)**	**Smokers(n=31 499)**
**16–24**				
**FEV**_**1**_**/FVC%**	86.9(86.7–87.2)*	86.2 (86.0–86.4)	88.0 (87.6–88.5)*	86.9 (86.7–87.3)
**FVC (l)**	4.907 (4.885–4.930)	4.920 (4.902–4.937)	3.520 (3.481–3.538)	3.560 (3.539–3.581)
**FVC%**	96.0 (95.6–96.4)*	96.9 (96.6–97.3)	93.4 (92.7–94.0)	94.8 (94.3–95.3)
**25–34**				
**FEV**_**1**_**/FVC%**	86.0 (85.9–86.1)*	85.3 (85.2–85.4)	87.2 (87.1–87.4)*	86.4 (86.3–86.6)
**FVC (l)**	4.873 (4.864–4.884)	4.875 (4.865–4.883)	3.540 (3.527–3.553)	3.563 (3.553–3.573)
**FVC%**	97.4 (97.7–98.0)	97.7 (97.6–97.9)	98.1 (97.8–98.4	98.3 (98.0–98.5)
**35–44**				
**FEV**_**1**_**/FVC%**	85.6 (85.5–85.7)*	84.5 (84.4–84.6)	86.3 (86.1–86.5)*	85.1 (84.9–85.2)
**FVC (l)**	4.623 (4.613–4.633)	4.556 (4.547–4.565)	3.399 (3.386–3.412)	3.378 (3.367–3.389)
**FVC%**	98.8 (98.7–99.0)*	97.4 (97.2–97.6)	102.2 (101.8–102.5)	101.4 (101.1–101.7)
**45–54**				
**FEV**_**1**_**/FVC%**	85.3 (85.2–85.4)*	83.2 (83.1–83.3)	85.9 (85.8–86.1)*	83.9 (83.7–84.1)
**FVC( l)**	4.224 (4.214–4.233)	4.088 (4.077–4.099)	3.075 (3.063–3.087)	3.036 (3.022–3.049)
**FVC%**	97.7 (97.5–97.9)*	94.9 (94.7–95.1)	102.8 (102.5–103.2)*	100.2 (99.7–100.6)
**55–64**				
**FEV**_**1**_**/FVC%**	84.2 (84.1–84.4)*	81.6 (81.3–81.8)	85.5 (85.2–85.8)*	83.7 (83.2–84.2)
**FVC (l)**	3.746 (3.733–3.759)	3.588 (3.570–3.605)	2.710 (2.692–2.729)	2.654 (2.623–2.685)
**FVC%**	95.6 (95.3–95.9)*	91.6 (91.2–92.0)	101.8 (101.2–102.5)*	98.9 (97.8–100.0)
**>64**				
**FEV**_**1**_**/FVC%**	83.4 (82.5–84.3)*	80.2 (78.3–82.2)	85.8 (84.0–87.6)	79.4 (71.8–86.9)
**FVC (l)**	3.230 (3.112–3.350)	3.480 (3.405–3.554)	2.396 (2.293–2.499)	2.350 (1.955–2.744)
**FVC%**	88.9 (85.6–92.2)	92.9 (91.2–94.6)	101.2 (97.2–105.1)	86.7 (79.9–93.6)

Values are means and 95% confidence intervals.

* Statistically significant differences between smokers and nonsmokers for males and females (*t* test for independent groups, with *P* < 0.001).

FVC(l):*Forced vital capacity in liters*.

FVC(%):*Forced vital capacity expressed as a percentage respect to normal values.*.

FEV_1_: *Forced volume in 1 second in liters.*

FEV_1_/FVC%: *The ratio of FEV*_*1*_*to FVC expressed as a percentage.*

When presence of bronchial obstruction reflected by FEV_1_% was compared between age ranges, sexes, BMI ranges, and WBC count, we detected significant differences for male smokers between the ages of 35 and 64 years, regardless of BMI, and for female smokers with a BMI <30 between the ages of 44 and 54 years (Table 6).

**Table 6:** **White blood cell count (cells/ml) and FEV**_**1**_**% in smokers**

		**Male smokers**			**Female smokers**		
**Age (years)**	**FEV**_**1**_**(% )**	**BMI≥30**	**25≤ BMI <30**	**BMI <25**	**BMI≥30**	**25≤ BMI <30**	**BMI <25**
**16–24**	**≥80**	7595(1824)	7072(1735)	6895(1746)	8121(1978)	7458(1849)	7024(1887)
	**≥50 <80**	9102(2281)	7855(1992)	6649(1967)	10300(-)	8764(2128)	7128(1780)
	**<50**	—	7400(-)	7430 (395)	5650(-)	—	7517 (4931)
**25–34**	**≥80**	7623(1873)	7136(1848)	6923(1852)	8014(1967)	7252(1887)	6821(1819)
	**≥50 <80**	8124(1876)	7543(1889)	6988(2237 )	7701(1810)	7447(1619)	7142(1959)
	**<50**	9615(2211)	7096(1596)	7300(2937)	8367(1760)	8050(1906)	7538(1922)
**35–44**	**≥80**	7595(1944)	7188(1954)	7083(2094)	7732(1999)	7138(1867)	6778(1876)
	**≥50 <80**	8216(2208) *	7613(1925)	7768(2468) *	7819(1723)	6926(1780)	7284(2321)
	**<50**	7965(1497)	7993(2102)	9589(3245)	9480(1584)	6250(540)	7772(2286)
**45–54**	**≥80**	7386(1859)	7134(1955)	7265(2168)	7108(1800)	6747(1785)	6530(1798)
	**≥50 <80**	8240(2161) *	7872(2090) *	8602(2419) *	8042(2080)	7696(1724) *	7682(1867) *
	**<50**	7912(2120)	8269(2255)	8333(2508)	8258(1756)	7696(2236)	6831(1090)
**55–64**	**≥80**	7141(1732)	6983(1815)	7087(1984)	6646(1671)	6305(1551)	6139(1616)
	**≥50 <80**	8031(2150) *	7736(8042) *	7720(1853) *	6607(1505)	7462(2085)	6781(2393)
	**<50**	7649(1779)	7704(1687)	8291(1937)	5863(1244)	6192(1922)	6598(1642)

Values are means (SD).

*Median test for white blood cell count in male and female smokers. Comparisons are between three levels of airway obstruction reflected by FEV_1_%. (P < 0.001)

BMI: *Body mass index (kg/m*^*2*^*).*

FEV_1_(%): *Forced vital capacity expressed as a percentage respect to normal value*.

ANCOVA confirmed the effect of age, sex, smoking and BMI on log (WBC) count demonstrating that the weight of the variable smoking was greater than all the other variables. In the established model for the logarithm of WBC count were included the set of covariates given by smoking, sex, BMI and age. All variables were statistically significant. About smoking, smokers have statistically higher levels of WBC than not smokers (estimated coeff = 0.176, SE = 0.001). Abou sex, males have statistically higher levels of WBC than females (estimated coeff = 0.022, SE = 0.001).

For quantitative covariates, BMI and age, the results obtained from the estimated model were: BMI-estimated coeff = 0.0001(SE = 0.00005) and age-estimated coeff = 8.494 (SE = 0.003).

## Discussion

This study provides clear confirmation that WBC count rises with smoking, even in a healthy, employed population. The count was higher in smokers by 1000–1300 cells/ml across a broad range of ages for both men and women and across a broader span of ages for men. Spirometric markers of lung function were also lower in healthy smokers.

The relation between smoking and WBC count has been reported previously [4,12-15]. Zalokar and colleagues [4], on studying more than 7000 men employed in public administration in Paris, found that a higher percentage of smokers had WBC counts over 6800 cells/ml. More recently, Smith and colleagues [12] demonstrated higher counts in a sample of 6902 male and 8405 female smokers. Elevated WBC counts play a role in processes that lead to cardiovascular diseases and increased risk of death. In one recent cohort study, mortality was higher in relation to a WBC count over 6000 cells/ml [13]. In an early prospective cohort study in which subjects were followed for 6.5 years, Friedman and colleagues [14,15] showed that smoking was associated with higher rates of myocardial infarcts and that WBC counts were much higher in smokers.

Age is also associated with WBC count. Although it was recently claimed that this marker decreases with age [13], we found that older male smokers had higher WBC counts, while levels in nonsmokers remained stable (Table 3), a finding that is consistent with a widely reported dose–response effect of smoking [4,16-22]. The observation of a lower WBC count in smokers who quit also points to the relevance of dose on effect. Roethig and colleagues [23] recently reported that WBC count decreased within the first three days of abstinence and that lower counts were sustained after a year of follow-up of former smokers. At five years, WBC counts approximated those of nonsmokers. In differential cell counts, elevated subpopulations of granulocytes and lymphocytes have also been observed in smokers [12,23-25]. However, it is important to note that BMI also affects WBC count, with obesity creating a proinflammatory state characterized by increases in C reactive protein and interleukin 6 concentrations as well as in WBC count [26,27]. In our study, ANCOVA identified BMI as a covariant of WBC count, as smokers in higher BMI strata were found to have elevated counts regardless of sex or lung function. Within age ranges, however, male smokers who had developed airflow limitation had still higher counts.

BMI and smoking have an inverse relationship [28]. Recently, Kauffman et al. showed that this relationship is moderated by sedentary conduct [29]. BMI was higher in larger sedentary compared to lower sedentary smokers. In our work, while BMI was statistically significant, this difference was not clinically significant The health benefit of being a nosmoker possibly outways by far the extra risk that less than a 1 kg/m^2^ increase in BMI.

Finally, our study also demonstrated lower spirometric variables (FEV_1_% and FEV_1_/FVC%) in smokers and that WBC counts were more sharply elevated in smokers with evident airflow limitation. This association between the development of bronchial obstruction and the degree of systemic inflammation reflected by total WBC count is clinically significant and consistent with the findings of Gan and colleagues [30,31]. These authors studied more than 7000 adults (>40 years of age) during a national nutritional study that collected data on several markers of inflammation, observing that counts and concentrations were high in smokers and that elevation was related to FEV_1_%. Such findings, like ours, indicate that the effects of smoking clearly extend beyond the well-known repercussions on the respiratory system to include low-grade systemic inflammation, a known risk factor for health conditions that increase risk of mortality such as arterial sclerosis, heart failure, and reduced cerebral blood flow. That we found that even the young smokers in our study were affected underlines the importance of initiating preventive measures as soon as an individual acquires the smoking habit.

The study’s main limitation was the unavailability of information about smoking duration and dose (packet-years), which would have given us greater understanding of the dose–response effect of smoking on WBC count. On the other hand, the study’s strength resides in its large sample size and an extensive age range.

In summary, this study in a healthy population undergoing a routine workplace check-up provides direct evidence of the proinflammatory effect of smoking, specifically elevated WBC counts. The link between smoking and elevated WBC count persisted even after stratification of the sample for age, sex, and BMI, which were also independent predictors. Finally, in this healthy population we observed a close tie between the development of airflow limitation and smoking. Although this study has provided clear evidence of a proinflammatory effect of smoking that begins early in healthy smokers, the cross-sectional design does not allow us to provide incidence rates that would indicate when disease processes begin to develop.

This research has been partially supported by a grant (MTM2009-10893) from the Ministry of Education of Spain.

## Competing interests

The authors declare that they have no competing interests.

## Authors’ contributions

JAFF, JMP, JVMA, ACM, AVF, AE, and JAGM participated in the elaboration and analysis of the data, as well as in the writing of the present manuscript. All authors have read and approved the final manuscript.
